# Supplementary material for: Family caregiver perspectives on strengths and challenges in the care of pediatric injury patients at a tertiary referral hospital in Northern Tanzania
Source: PLoS One. 2023 Dec 15;18(12):e0286836. doi: 10.1371/journal.pone.0286836 (PMC10723720; doi:10.1371/journal.pone.0286836)
Supplement: Attachment — Submitted filename: PLOSONE_Response to Reviewers.docx [file pone.0286836.s001.docx]

**Response to Reviewers:**

Journal Requirements:

When submitting your revision, we need you to address these additional requirements.

**1. Please ensure that your manuscript meets PLOS ONE's style requirements, including those for file naming. The PLOS ONE style templates can be found at**

[**https://journals.plos.org/plosone/s/file?id=wjVg/PLOSOne_formatting_sample_main_body.pdf**](https://journals.plos.org/plosone/s/file?id=wjVg/PLOSOne_formatting_sample_main_body.pdf) **and**

[**https://journals.plos.org/plosone/s/file?id=ba62/PLOSOne_formatting_sample_title_authors_affiliations.pdf**](https://journals.plos.org/plosone/s/file?id=ba62/PLOSOne_formatting_sample_title_authors_affiliations.pdf)

Thank you for this instruction. We have edited our manuscript to meet the style requirements per the templates.

**2. Thank you for stating in your Funding Statement:**

**"Fogarty International Center"**

**Please provide an amended statement that declares *all* the funding or sources of support (whether external or internal to your organization) received during this study, as detailed online in our guide for authors at** [**http://journals.plos.org/plosone/s/submit-now**](http://journals.plos.org/plosone/s/submit-now)**.  Please also include the statement “There was no additional external funding received for this study.” in your updated Funding Statement.**

**Please include your amended Funding Statement within your cover letter. We will change the online submission form on your behalf.**

**One or more authors are affiliated with the funder, but authors state that the funder had no role**

**Thank you for stating the following financial disclosure:**

**"EMK was supported in this work by the Fogarty International Center of the National Institutes of Health (D43 TW009337). The content is solely the responsibility of the authors and does not necessarily represent the official views of the National Institutes of Health. The funders had no role in study design, data collection and analysis, decision to publish, or preparation of the manuscript."**

Thank you for these instructions. The amended statement that declares all the funding or sources of support is as follows and has been edited as such in the manuscript page 23: “EMK was supported in this work by the Fogarty International Center of the National Institutes of Health (D43 TW009337) and the Eunice Kennedy Shriver National Institute of Child Health and Human Development (1K23HD112548-01). The content is solely the responsibility of the authors and does not necessarily represent the official views of the National Institutes of Health. The funders had no role in study design, data collection and analysis, decision to publish, or preparation of the manuscript. There was no additional external funding received for this study.”

**3. In your Data Availability statement, you have not specified where the minimal data set underlying the results described in your manuscript can be found. PLOS defines a study's minimal data set as the underlying data used to reach the conclusions drawn in the manuscript and any additional data required to replicate the reported study findings in their entirety. All PLOS journals require that the minimal data set be made fully available. For more information about our data policy, please see** [**http://journals.plos.org/plosone/s/data-availability**](http://journals.plos.org/plosone/s/data-availability)**.**

**Upon re-submitting your revised manuscript, please upload your study’s minimal underlying data set as either Supporting Information files or to a stable, public repository and include the relevant URLs, DOIs, or accession numbers within your revised cover letter. For a list of acceptable repositories, please see** [**http://journals.plos.org/plosone/s/data-availability#loc-recommended-repositories**](http://journals.plos.org/plosone/s/data-availability#loc-recommended-repositories)**. Any potentially identifying patient information must be fully anonymized.**

**Important: If there are ethical or legal restrictions to sharing your data publicly, please explain these restrictions in detail. Please see our guidelines for more information on what we consider unacceptable restrictions to publicly sharing data:** [**http://journals.plos.org/plosone/s/data-availability#loc-unacceptable-data-access-restrictions**](http://journals.plos.org/plosone/s/data-availability#loc-unacceptable-data-access-restrictions)**. Note that it is not acceptable for the authors to be the sole named individuals responsible for ensuring data access.**

**We will update your Data Availability statement to reflect the information you provide in your cover letter.**

Thank you for these instructions. There are legal restrictions to sharing our data publicly as the data for this manuscript is covered under a data sharing agreement and is owned by KCMC in Tanzania. Here is our updated Data Availability statement: “The data for this manuscript is covered under a data sharing agreement, and thus we are unable to openly share these data. For data access requests, please contact our non-author KCMC representative Gwamaka William at [gwamakawilliam14@gmail.com](mailto:gwamakawilliam14@gmail.com).”

**4. Please amend either the abstract on the online submission form (via Edit Submission) or the abstract in the manuscript so that they are identical.**

Thank you for catching this error. We have amended the abstract on the online submission form so that it is identical to the abstract in the manuscript.

**5. Please upload a new copy of Figure 1 as the detail is not clear. Please follow the link for more information:** [**https://blogs.plos.org/plos/2019/06/looking-good-tips-for-creating-your-plos-figures-graphics/**](https://blogs.plos.org/plos/2019/06/looking-good-tips-for-creating-your-plos-figures-graphics/)**"** [**https://blogs.plos.org/plos/2019/06/looking-good-tips-for-creating-your-plos-figures-graphics/**](https://blogs.plos.org/plos/2019/06/looking-good-tips-for-creating-your-plos-figures-graphics/)

Thank you for this instruction. A larger copy of Figure 1 has been uploaded that has clear detail.

**Review Comments to the Author**

**Reviewer #1: The study "Caregiver perspectives on strengths and challenges in the care of pediatric injury patients at a tertiary referral hospital in Northern Tanzania" aims to understand the experiences and views of caregivers who provide care to pediatric injury patients at a tertiary referral hospital in Northern Tanzania. The study explore the strengths and challenges faced by caregivers in providing care to these patients. The study could have had the possibility provide insights into the healthcare system and identify areas for improvement.**

Thank you for this summary. However, we believe this reviewer may have misunderstood the population that we interviewed, as we interviewed *family member* caregivers of pediatric injury patients, not the medical caregivers *who provide care* to the patients in the hospital. In order to make this more clear, we have added “family member” to caregivers in the abstract, introduction, methods, and conclusions. We have also revised the title to “Family caregiver perspectives on strengths and challenges in the care of pediatric injury patients at a tertiary referral hospital in Northern Tanzania.”

**However, the study uses only qualitative methods to collect data from caregivers and limited sample size (i.e. 30 caregivers).**

Thank you for noting this. Yes, this was a study using only qualitative methods. Regarding our sample size, we have provided more information regarding why this sample size was chosen on pages 7, lines 268-274: “The plan to recruit 30 caregivers was informed by team-based discussions on when thematic saturation was most likely to occur. Previous work with in-depth interview data found that thematic saturation was present after 12 interviews. However, in order to adequately represent our varied pediatric injury population, we felt that a larger sample size of 30 would be more likely to achieve saturation. We thus approached caregivers of children of varying ages, mechanisms of injury, and injury types. Attention was given to interviewing caregivers who would be good informants.” In addition, we did reach saturation as anticipated, which has been added to the end of the data analysis section in the methods in line 331.

**The conclusion is erratic and wrote haphazardly.**

Thank you for this feedback. We have edited the conclusion so that it is more clear and focused.

**Too many ambiguous and unnecessary abbreviations were used. It does not provide any unique or intriguing aspect that would make readers eager to explore further.**

Thank you for these comments. We have gone through and removed abbreviations that were unnecessary such as WHO, PI, TBI, HICs, HIV, SSA. We have spelled these out instead. We are now left with only five abbreviations that are used frequently throughout the manuscript.

**Additionally, the methodology or approach used in gathering the caregiver perspectives, which leaves the reader wondering about the reliability and validity of the data. Without this information, it becomes difficult to fully grasp the scope and depth of the study.**

Thank you for this comment that our methodology needs to be strengthened. We have strengthed our methodology by clearly stating how we chose the sample size (lines 268-274), how we selected participants (lines 272-274), and elaborated more on the study procedure including the piloting (lines 289-292). These methodology clarifications allow the reader to adequately assess the study’s internal validity.

**The mention of a specific location, "Northern Tanzania," may limit the relevance and applicability of the findings to a wider audience. Readers outside of this region might view the study as having limited generalizability to their own settings, thereby diminishing its overall impact.**

Thank you for this point. It is true that doing a study in one specific location may limit the relevence and applicability of the findings to a wider audience; however, we did state that this is a single center study and thus conclusions must be interpreted as such. While some of our study’s fundings may be site-specific, many systems issues will have relevance to other settings. This has been made clearer by adding the following to the Limitations section, lines 686-690: “An additional limitation is that this study was done at a single tertiary referral hospital in Northern Tanzania, and extrapolating conclusions to other sites must be done with caution. While some findings may be site-specific, we expect that similar system-level issues exist in other tertiary hospitals in resource-denied settings.”

**Furthermore, It does not indicate how the findings could be utilized to improve the care of pediatric injury patients or address the challenges faced by caregivers.**

Thank you for this comment. In the first paragraph of our discussion, we give three examples of how the challenges identified in our findings could be addressed in lines 587-591: “The challenges identified by the caregivers in our study will be the foundation for future interventions including the introduction of a patient liaison who serves to update patient caregivers on their patient’s status and the plan of care, ensuring all direct communication is in or translated to the caregivers’ preferred language, and standardizing instructions regarding discharge and follow-up.” We hope that the additional changes made, incorporating the reviewers’ thoughtful feedback, help make the points made in this section of the discussion more clear.

**In summary, the study lacks originality, fails to provide crucial information about the methodology, limited generalizability, and lacks emphasis on potential outcomes. These shortcomings diminish its effectiveness in capturing readers' interest and communicating the significance of the study.**

Thank you for this summary of your review. These peer-reviewed data will be the foundation for future interventions, which has been added to the Discussion line 588 and Conclusion line 697. Even though this was a single site study, the methodology used and themes identified are very relevant to many settings in Sub-Saharan Africa and other LMICs in general, which has been added to the Conclusion lines 698-700 along with a citation that demonstrates this (reference 40). We hope that by addressing your comments above we have made this study more effective in capturing readers’ interest and communicating the significance of the study.

**Reviewer #2: I am pleased to provide a review of the intriguing paper titled "Caregiver perspectives on strengths and challenges in the care of pediatric injury patients at a tertiary referral hospital in Northern Tanzania." The entire paper is well-written; however, the revised version should address the following comments.**

Thank you for this compliment.

**1. The introductory section appears to be insufficient in length. It is recommended to expand the content by incorporating additional paragraphs and introducing new references that are relevant to the study.**

Thank you for this feedback. We have expanded the content of the introduction as suggested by adding an additional paragraph and introducing new references relevant to the study on page 5, lines 219-229.

**2. Please provide the list of abbreviations.**

Thank you for this instruction. We have provided the list of abbreviations after the title page and before the abstract.

**3.Please ensure that the reference number and date for your ethical approval are included.**

Thank you for catching this omission. We have added reference numbers and dates for all three ethical approvals in the methods section lines 262-263.

**4. Please provide more detailed explanations regarding the data availability statement.**

Thank you for this request. There are legal restrictions to sharing our data publicly as the data for this manuscript is covered under a data sharing agreement and owned by KCMC in Tanzania. Here is our updated Data Availability statement: “The data for this manuscript is covered under a data sharing agreement, and thus we are unable to openly share these data. For data access requests, please contact our non-author KCMC representative Gwamaka William at [gwamakawilliam14@gmail.com](mailto:gwamakawilliam14@gmail.com).”

**Reviewer #3: In this qualitative study, the authors aimed to identify the strengths and challenges to care of pediatric injury patients at a tertiary hospital. The methodology and the results are well described. However the sample size is the major limitation of this study.**

Thank you for this summary. Regarding our sample size, we have provided more information regarding why this sample size was chosen on pages 6-7, lines 268-274: “The plan to recruit 30 caregivers was informed by team-based discussions on when thematic saturation was most likely to occur. Previous work with in-depth interview data found that thematic saturation was present after 12 interviews. However, in order to adequately represent our varied pediatric injury population, we felt that a larger sample size of 30 would be more likely to achieve saturation. We thus approached caregivers of children of varying ages, mechanisms of injury, and injury types. Attention was given to interviewing caregivers who would be good informants.” In addition, we did reach saturation as anticipated, which has been added to the end of the data analysis section in the methods in line 331.
