# Supplementary material for: Family caregiver perspectives on strengths and challenges in the care of pediatric injury patients at a tertiary referral hospital in Northern Tanzania
Source: PLoS One. 2023 Dec 15;18(12):e0286836. doi: 10.1371/journal.pone.0286836 (PMC10723720; doi:10.1371/journal.pone.0286836)
Supplement: Attachment — Submitted filename: PLOSONE_Response to Reviewers 2.docx [file pone.0286836.s002.docx]

**Response to Reviewers/Editors:**

1. Please specifically state when saturation was achieved. Your statement at the line 191 does not tell whether this was achieved at exactly the 30^th^ IDI or before and at which IDI.

**Thank you for this question. While the analysis was iterative, there was a timelag from the data collection to transcription and translation, and then to data analysis. As such, we completed the established target of 30 IDIs. In analyzing the data, we confirmed that data saturation was reached at 30, and therefore we decided it was not necessary to complete additional interviews. This was clarified in lines 209-210 of the revised manuscript.**

2. Explain further your selection of participants. It is essential to elaborate more on your inclusion/exclusion criteria and especially the rationale to exclude caregivers whose patients died, the impact of this exclusion on your findings.

**Thank you for this instruction. The characteristics of participants identified is described in lines 139-155. We have elaborated on selection of participants by including information that each pediatric injury patient was enrolled in a pediatric injury registry on arrival to KCMC, and during registry completion caregivers were identified who would be good informants for an interview (lines 143-145). In addition, text was added to lines 154-155 that attention was given to interviewing caregivers who would be good informants, in that when completing the registry they appeared to be comfortable and forthcoming speaking about their child’s healthcare experience. In this revision we also clarified that death of the child in the hospital was not an exclusion criteria from participating in the study, and text has been added to make this clear in lines 141-142, 144, 168, and 178-179.**

3. It is observed that you have had the same number (3) of themes for strengths as well as for challenges, at EMD, wards, and at discharge. Please explain how this has happened, whether it was a mere coincidence or a specific reason and planned ahead of time.

**Thank you for this inquiry. The identification of 3 themes for strengths and challenges at all phases of hospitalization did happen by mere coincidence. In this revision we have identified it as coincidence in lines 216-218 to address it up front.**

4. Lastly, read carefully your manuscript to make sure all your statements are correctly spelt. E.g., the statement: “Authors did not have access to information that could identify individual participants during or after data collection” [Lines 185-186] is in contradiction with the subsequent statement: “IDIs were conducted by authors GN and IA, who were research assistants at the time of the study” [Lines 189-190].

**Thank you for pointing out this contradiction. We have corrected this in the revised manuscript. We have added “Other authors…” to distinguish that authors other than the research assistants did not have access to information that could identify individual participants during or after data collection. The remainder of the manuscript was carefully read to ensure all our statements are correctly spelled, and small wording changes were made as necessary throughout.**

5. On the lines 336-337, you are neutrally talking about a patient but then assigned him/her a female gender. “…If the patient is feeling, …..she can get anti pain”.

**Thank you for pointing this out. The "she" was in-fact a typo and has been corrected to "he," but in this instance the discordance between the neutral and gendered pronouns was taken from a quote from a parent, who is referring to his male child who was a pediatric injury patient. The parent included both neutral and gendered pronouns in their quote and thus we kept it this way as to not alter their words.**
